# Case report: Pulmonary Ewing sarcoma disguised as non-small cell lung cancer

**DOI:** 10.3389/fonc.2024.1449119

**Published:** 2024-11-07

**Authors:** Mary E. Carter, Alessia Benegiamo-Chilla, Linus D. Kloker, Nikolas Paulsen, Vlatko Potkrajcic, Frank Paulsen, Attila Nemeth, Volker Steger, Martin Schulze, Saskia Biskup, Katrin Benzler, Stephan Singer, Ulrich M. Lauer, Lars Zender, Christoph K. W. Deinzer

**Affiliations:** ^1^ Department of Medical Oncology and Pneumology, Medical University Hospital, Tübingen, Germany; ^2^ Department of Radiation Oncology, University Hospital, Tübingen, Germany; ^3^ Department of Thoracic and Cardiovascular Surgery, University Hospital, Tübingen, Germany; ^4^ Zentrum für Humangenetik Tübingen, Tübingen, Germany; ^5^ CeGaT GmbH, Center for Genomics and Transcriptomics, Tübingen, Germany; ^6^ Institute of Pathology and Neuropathology, University Hospital Tübingen, Tübingen, Germany; ^7^ University of Tübingen, iFIT Cluster of Excellence (EXC2180) “Image-Guided and Functionally Instructed Tumor Therapies”, Tübingen, Germany; ^8^ German Cancer Research Consortium (DKTK), Partner Site Tübingen, German Cancer Research Center (DKFZ), Heidelberg, Germany

**Keywords:** case report, Ewing sarcoma, pulmonary Ewing sarcoma, chemotherapy, molecular pathology, *EWSR1::FLI1*, tumor resection

## Abstract

Ewing sarcoma is the second most common primary malignant bone cancer in children and adolescents. This rare type of cancer is characterized by its high malignancy and therefore high risk of metastases. Typically, Ewing sarcomas originate from bones. However, extraosseous Ewing sarcoma such as pulmonary Ewing sarcoma can also be found. In this case report, we present a 55-year old male patient who was initially diagnosed with non-small cell lung cancer at his local district hospital. However, the diagnosis was changed to one of pulmonary Ewing sarcoma after subsequent histopathological and molecular pathological analysis performed in a reference pathology laboratory. After patient referral to a certified (according to the German Cancer Society) high-volume sarcoma center, multimodal chemotherapy was initiated based on recently published clinical data as opposed to the more commonly used treatment regimen in Europe. The patient responded well to treatment and underwent a complete surgical tumor resection followed by radiotherapy. In summary, this case report highlights the importance of a rigorous and timely histopathological examination of biopsy samples by a specialized cancer center to enable a correct diagnosis of the cancer type. Additionally, molecular pathology plays a crucial part in this analysis and allows the necessary differentiation between cancer types. Up to now, there is no international treatment guideline available for the treatment of Ewing sarcoma. Patients should be referred to specialist centers to allow the best possible treatment of the cancer type in view of current published clinical data. In the case of Ewing sarcoma, and in accordance with the most recent research, patients should be treated with vincristine, doxorubicin and cyclophosphamide plus ifosfamide and etoposide in combination with local treatment such as surgery and/or radiotherapy because this has been demonstrated to be the more effective therapy.

## 1 Introduction

### 1.1 Ewing sarcoma

The Ewing’s sarcoma family of tumors (ESFT) includes four main types of cancer: osseous Ewing sarcoma, primary neuroectodermal tumors, Askin tumor (Ewing sarcoma originating from the chest wall) and extraosseous Ewing sarcoma such as pulmonary Ewing sarcoma (1, 2).

Although the average annual incidence in the population is only 2.9 per million, Ewing sarcoma is, nevertheless, still the second most common primary malignant bone cancer in children and adolescents (2). Patients mostly present in their second to third decade of life (3, 4).

This rare type of cancer is characterized by its high malignancy and therefore high risk of metastases (3). Despite multimodal treatment, long-term survival in metastatic disease occurs in only 20–25% of patients. Metastases are predominantly present in the lungs (70–80%) and bone/bone marrow (40–45%) and are associated with a dismal prognosis. In addition, recurrent disease is observed in 30–40% of patients with primary non-metastatic disease, increasing to 60–80% for patients with metastatic disease at initial diagnosis (3).

An important part of the diagnostic workflow is histopathological characterization. Ewing sarcomas are characterized by a solid growth pattern with monomorphic small cells displaying round nuclei (5). Most Ewing sarcomas stain positive with immunohistochemical testing for cluster of differentiation (CD) 99 (5). However, this marker is not specific for Ewing sarcoma (3). A definitive diagnosis of Ewing sarcoma is only possible by molecular pathology. Ewing sarcoma is characterized by an aberrant gene fusion. The rearrangement often includes the *Ewing sarcoma breakpoint region 1* (*EWSR1*) which in most cases is joined with *Friend leukemia integration 1* (*FLI1*) (6). The onco-fusion gene *EWSR1::FLI1* can be detected by using a fluorescent *in-situ* hybridization (FISH)-based method and/or reverse transcriptase polymerase chain reaction (RT-PCR) detection (3, 7). In addition, the fusion gene can be detected with DNA or RNA sequencing. FISH-based detection is widely used for diagnostic purposes. However, in difficult cases RT-PCR is performed in addition for reliable diagnosis (8). In accordance with the World Health Organization the testing for molecular translocation is a requirement for diagnosis of Ewing sarcoma (9, 10).

Patients with pulmonary Ewing sarcoma often present with a few symptoms that mimic pneumonia with fever and dyspnea (11). As a consequence, further investigation is conducted with diagnostic imaging such as by computed tomography (CT). These scans of the chest often reveal a single well-defined mass with an inhomogeneous appearance. Calcifications and pleural effusions can also be seen. In some cases, the mass can extend to the chest wall and mediastinum with possible invasion of surrounding structures (11–13).

### 1.2 Therapeutic approaches

Therapeutic approaches for Ewing sarcoma combine chemotherapy, surgery and radiotherapy, while interdisciplinary sarcoma tumor boards help to facilitate decision making for optimal patient treatment (14). Importantly, there has been no successful introduction of new drugs for Ewing sarcoma in the last 40 years (3). A combination of surgery with neoadjuvant and adjuvant chemotherapy is the standard of care due to the high risk of metastases (3, 15). Radiotherapy (RT) is an important additional treatment pillar for the treatment of Ewing sarcoma as this cancer type has been found to be sensitive to RT (16). RT can be utilized in addition to surgery or as definitive treatment for local control of inoperable tumors.

The therapeutic approach to patients with Ewing sarcoma usually consists of a combination of different chemotherapeutic agents. Currently, there is no internationally standardized chemotherapeutic treatment for Ewing sarcoma. The regimen widely used in Europe according to the EURO-EWING 99 trial combines induction chemotherapy (vincristine, ifosfamide, doxorubicin und etoposide [VIDE] before local procedures) with consolidation chemotherapy according to risk (vincristine, actinomycin D, and ifosfamide or cyclophosphamide [VAI or VAC]). Alternatively, high-dose busulfan and melphalan can be used instead of consolidation chemotherapy for localized disease with preselected high-risk factors which leads to an improvement in event-free survival and overall survival (17).

The treatment regimen used mainly in the US is based on the Children’s Oncology Group AEWS0031 trial (18). The induction therapy consists of alternating cycles of vincristine, doxorubicin and cyclophosphamide (VDC) with ifosfamide and etoposide (IE) once every two weeks. The subsequent consolidation therapy after surgery includes alternating cycles of IE and vincristine and cyclophosphamide (VC). The cycles during consolidation therapy have been shown to be more effective when administered once every 2 weeks (18).

A recent open-label randomized phase 3 trial (EE2012) directly compared the two therapeutic regimens mentioned above. The trial managed to recruit a total of 640 patients between the ages of 2 and 49 years and allocated 320 patients to each of two groups. Group 1 was treated with the European treatment regimen. Group 2 was treated according to the American protocol. The primary outcome measured was event-free survival. At 3 years, event-free survival was 67% for patients receiving VDC and IE as opposed to 61% for patients treated with VIDE (HR 0.71 [95% 0.55-0.92]). Overall, the results showed a higher effectivity, reduced toxicity and shorter duration of treatment. The trial concluded that the dose-intensive chemotherapy with VDC and IE is more beneficial for patients newly diagnosed with Ewing sarcoma and should therefore be used as the standard of care (15).

### 1.3 Case highlights

In this case report we describe a 55-year old male patient who was initially diagnosed with non-small cell lung cancer (NSCLC) at his local district hospital. However, the diagnosis was changed to one of pulmonary Ewing sarcoma after subsequent histopathological and molecular pathological analysis in a reference pathology laboratory. After patient referral to a certified (according to the German Cancer Society DKG) high-volume sarcoma center, multimodal chemotherapy was initiated based on recently published clinical data as opposed to the more commonly used treatment regimen in Europe. The patient responded well to treatment and underwent complete surgical tumor resection followed by radiotherapy to reduce the risk of tumor relapse.

## 2 Case report

### 2.1 Patient history

**Figure 1:**
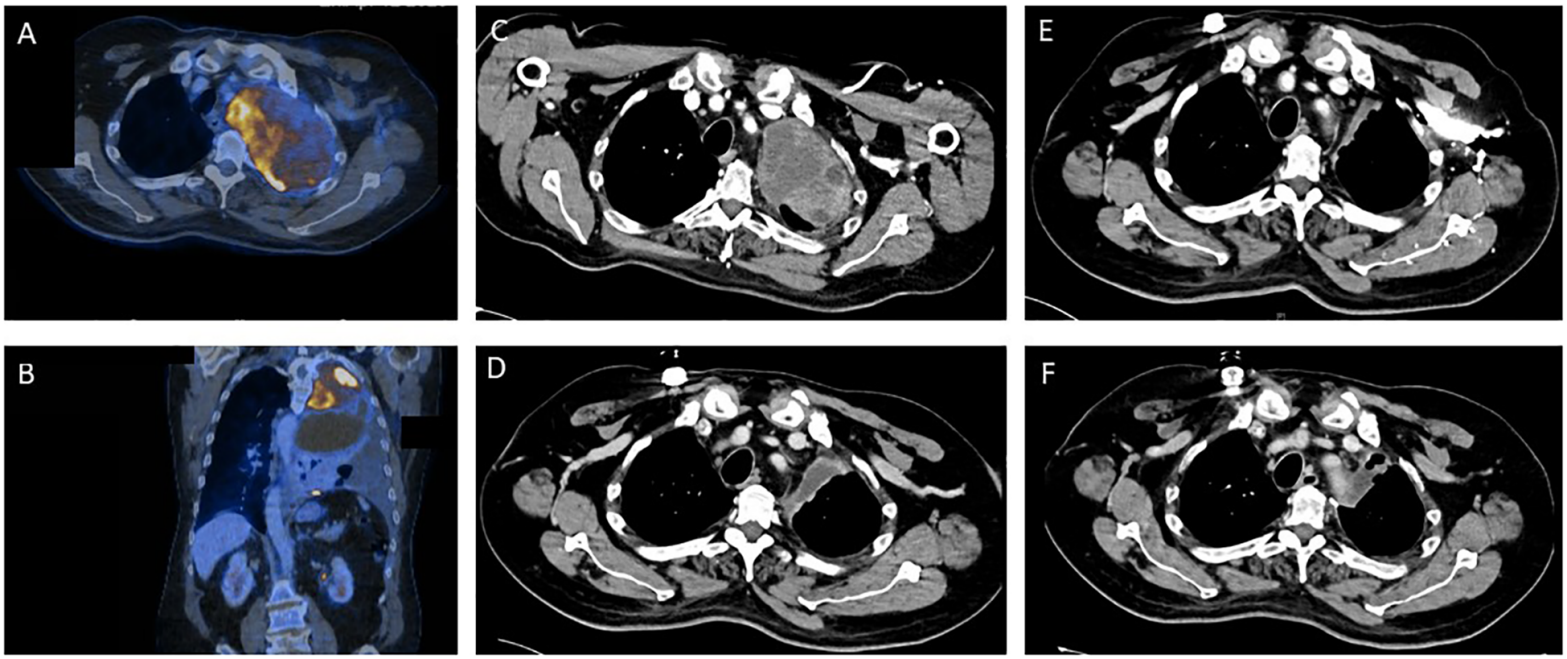
Tumor regression as depicted by sequential contrast enhanced computed tomography (CT) and positron emission tomography (PET) before and during the various stages of treatment for Ewing sarcoma. **(A, B)** Pre-therapeutic PET/CT imaging. **(C)** CT imaging prior to start of Ewing sarcoma chemotherapy. **(D)** CT Imaging after 2.5 cycles of induction chemotherapy. **(E)** CT Imaging after induction chemotherapy. **(F)** Post-operative CT imaging.

A 55-year-old man presented with coughing and dyspnea to his general practitioner. Two months after symptom onset the patient was referred to his local district hospital due to his worsening symptoms. A CT scan of the chest revealed a large pulmonary mass (14 cm x 9.5 cm) in the left upper lobe with infiltration of the thoracic wall, the left subclavian and vertebral artery (**Figures 1A, B**). The mass resulted in a shift of the mediastinum to the right. The patient had no previous relevant medical history or family history of cancer.

### 2.2 Diagnosis

A transthoracic biopsy of the mass in the left upper lobe led to an initial diagnosis of NSCLC by the institute of pathology of the district hospital. The initial primary tumor site and size (T), regional lymph node involvement (N) and possible distant metastatic spread (M), referred to as the TNM staging for NSCLC, was defined as cT4 cN2 cM1a (PLE), according to the Union for International Cancer Control (UICC) IV A. A pre-therapeutic PET-CT scan (**Figures 1A, B**) was performed externally.

Reference pathology for molecular analysis of lung cancer was routinely sent from the district hospital to the institute of pathology at a university hospital. These results showed a small cell, solid growing tumor with immunohistochemical staining positive for CD56 as well as CD99. Additional immunostainings showed that the tumor was negative for AE1/3 cytokeratins, CK 7, BerEP4, CD34, CK5-14, p40, SOX 10, NUT, protein S100, chromogranin, synaptophysin and thyroid transcription factor (TTF-1). Nucleal expression of INI-1 was found to be intact.

Subsequent molecular pathological analysis was performed with next-generation sequencing (NGS) based on the Archer PanST V2 panel. The results clearly demonstrated a *EWSR1::FLI1* (E7F5) fusion with breaking points at an RNA level after *ESWR1* exon 7 and before FLI1 exon 5, thereby resulting in a diagnosis of a Ewing sarcoma (7).

After this diagnosis the patient was referred to the certified high-volume sarcoma center at the University Hospital Tübingen. Our interdisciplinary sarcoma tumor board recommended a complete CT body scan. This scan (**Figure 1C**) revealed a slight reduction in tumor mass when compared to the initial PET-CT performed in the local district hospital as mentioned above. However, the extent of the pulmonary mass was still large and metastases were present in the thoracic wall.

### 2.3 Treatment

**Figure 2:**
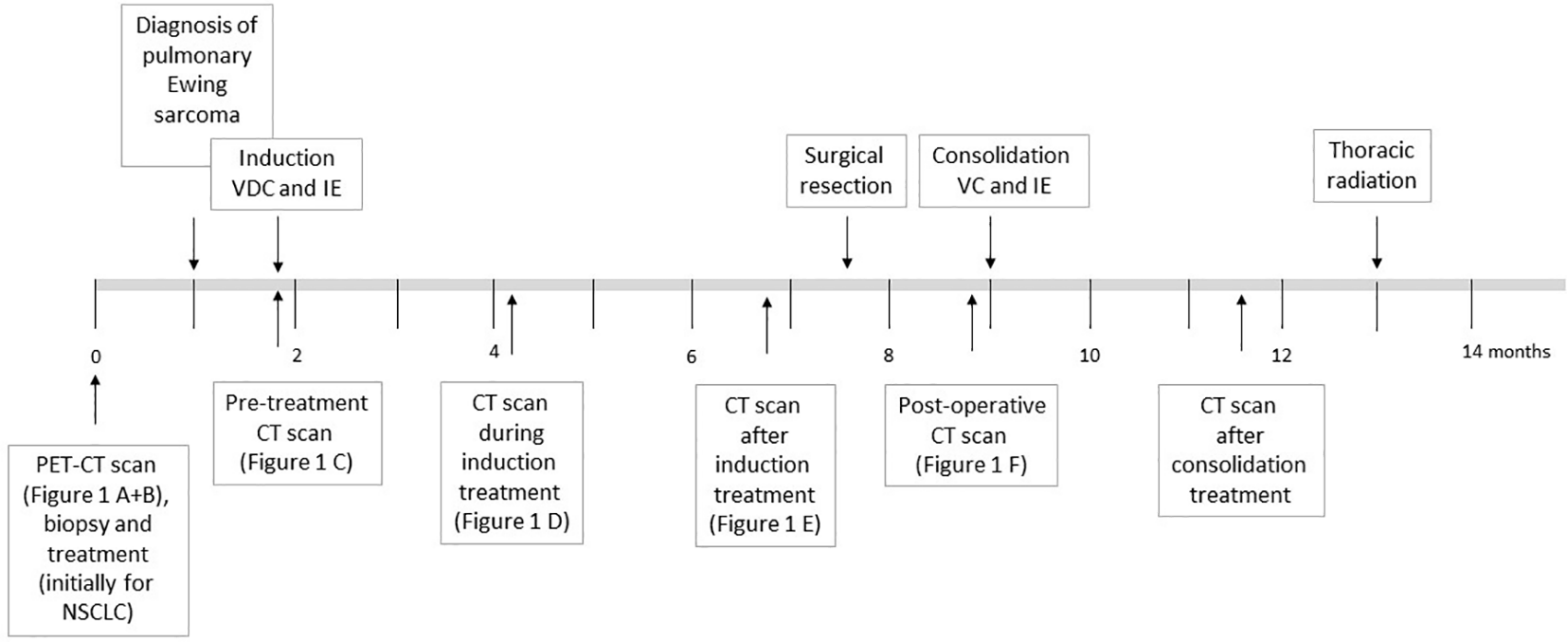
Overview of the chemotherapy treatment as described in the text for the patient diagnosed with Ewing sarcoma. The time in months since first hospital admission and diagnosis are shown.

**Figure 2** offers an overview of the treatment described below.

The patient received chemotherapy based on the initial diagnosis of NSCLC according to German guidelines for the treatment of NSCLC. Treatment consisted of two cycles of carboplatin (AUC 5), paclitaxel (175 mg/m^2^) and pembrolizumab (200 mg) due to the mediastinal shift and worsening dyspnea (19).

Shortly after the altered diagnosis and subsequent admission to the University Hospital Tübingen, our interdisciplinary sarcoma tumor board recommended initiation of a chemotherapeutic regimen according to the EWING 2012 trial (15). The patient was planned to receive 4.5 cycles of vincristine (1 day of 2 mg), doxorubicin (2 days of 37.5 mg/m^2^) and cyclophosphamide (1 day of 1200 mg/m^2^) followed by ifosfamide (5 days of 1800 mg/m^2^) and etoposide (5 days of 100 mg/m^2^). **Figure 1D** shows CT imaging after 2.5 cycles of induction chemotherapy.

After completion of 3 cycles of chemotherapy the patient underwent leukapheresis for a potentially necessary autologous blood stem-cell rescue.

The blood test showed a thrombocytopenia grade IV and a neutropenia grade IV. Consequently, the chemotherapeutic dose was reduced to 80% after 3.5 cycles. The patient completed the planned 4.5 cycles of chemotherapy. CT scans revealed a reduction in size of the pulmonary mass as well as no additional metastases (**Figure 1E**). Consequently, our interdisciplinary sarcoma tumor board recommended surgical tumor resection.

After completion of induction chemotherapy the patient underwent a left posterolateral thoracotomy with extended lobectomy of the left upper lobe with intrapericardial vessel resection. Additionally, a radical lymph node dissection was performed. The patient recovered well from surgery and there were no postoperative complications. The histopathological evaluation and molecular testing confirmed diagnosis of a *EWSR1::FLI1* fusion Ewing sarcoma and resulted in postoperative TNM classification of ypT2b, pN0 (0/4 LN), L0, V0, Pn0, R0. The vital residual tumor within the surgically removed tissue was described with 10%.

The pulmonary vital capacity increased during the preoperative chemotherapy from 1.06L to 1.60L. Due to initial exertional dyspnea, surgical resection was performed two months after completion of induction chemotherapy. After surgery the vital capacity further increased to 2.07L thereby resulting in increased cardiorespiratory exercise capacity for the patient. The postoperative CT scans revealed no pulmonary mass and no detection of metastases (**Figure 1F**). Additionally, no brain metastases could be detected in MRI scans and no bone marrow infiltration was found in bone marrow biopsy.

Postoperatively, the patient received 2.5 cycles of consolidation treatment with VC/IE. This treatment regimen included vincristine (1 day of 2 mg) and cyclophosphamide (1 day of 1200 mg/m^2^) followed by ifosfamide (5 days of 1800 mg/m^2^ in 80%) and etoposide (5 days of 100 mg/m^2^ in 80%). The post-therapeutic imaging revealed no residual tumor burden.

After surgery and postoperative consolidation chemotherapy, the patient will undergo radiotherapy of the operative field to reduce the risk of tumor relapse.

### 2.4 Molecular genetic analysis

**Figure 3:**
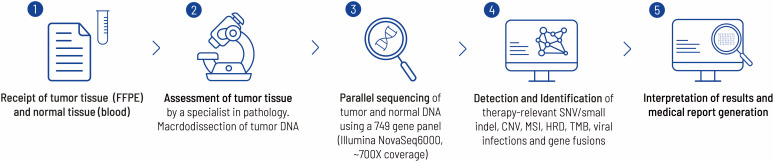
This figure highlights the work flow from sample receipt to the final medical report at the Centre for Human Genetics, Tübingen. Each FFPE block is examined by a pathology specialist before sequencing to verify the tumor entity and check for suitability with regard to tumor content. In the present case, a histological tumor content of 20% was determined. The bioinformatics pipeline has already been described elsewhere (26).

**Figure 4:**
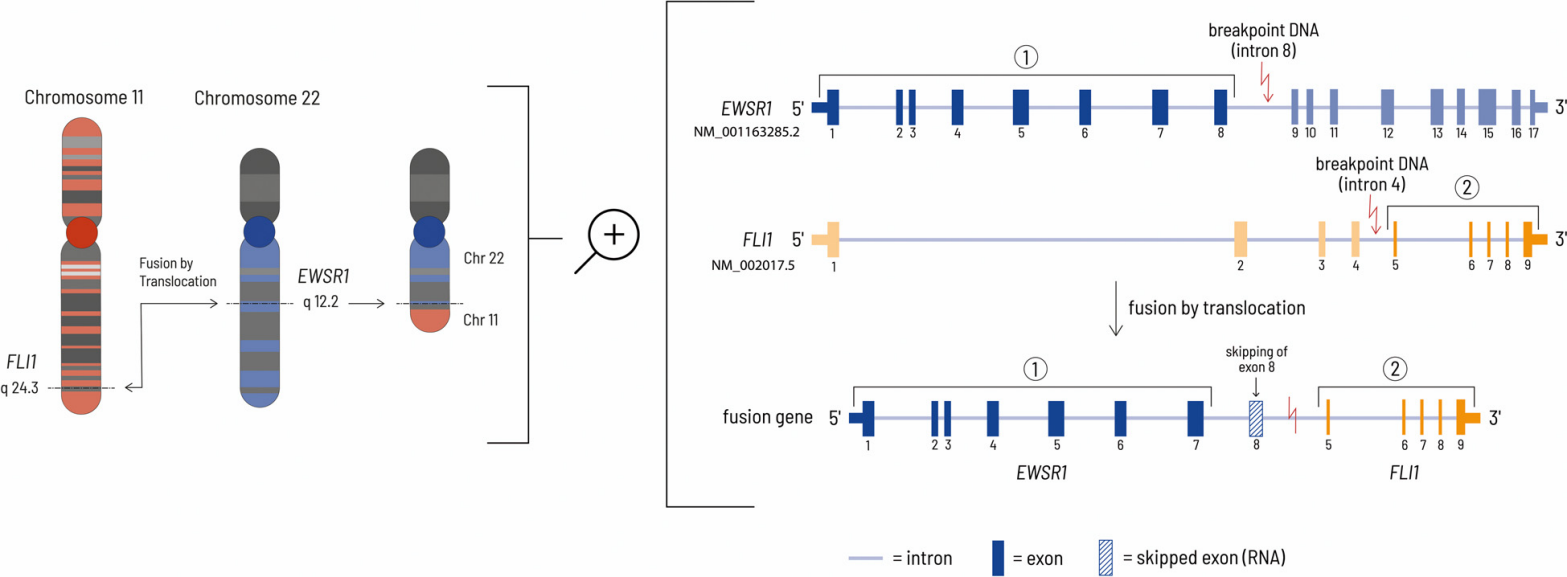
The left side of the figure shows the translocation event leading to the *EWSR1-FLI1* fusion gene at the chromosomal level. In this case, the genomic breakpoints are located at chr22:29685464 - chr11:128643985 (HG19 reference genome). The right side of the figure shows the details at the gene level. Although the genomic breakpoint in the EWSR1 gene is located after exon 8, the eighth exon of the *EWSR1* gene is removed during mRNA processing by HNRNPH1, thereby resulting in an inframe fusion protein consisting of the first 7 *EWSR1* exons and exons 5-9 of the *FLI1* gene (27, 28).

The molecular genetic analysis was performed by the Center for Human Genetics in Tübingen. Tissue obtained from the initial biopsy of the lung and from surgically resected tissue was analyzed (**Figures 3**, **4**). **Supplementary Table 1** offers an overview of the full gene list and list of biomarkers routinely described in the panel.

The surgically resected tissue included sufficient tumor cells to allow reliable results. NGS of the DNA from the biopsy tissue detected a *EWSR1::FL1* fusion gene, which confirmed the initial diagnosis of Ewing sarcoma. In addition, an oncogenic mutation of the *human telomerase reverse transcriptase* (*TERT*) gene was found. The tumor displayed a low tumor mutational burden with 0.4 Var/Mb. Further analysis could not detect microsatellite instability or a deficit in homologous recombination.

A *ESWR1* fusion gene is typical for patients with Ewing sarcoma (20). The gene fusion consists of the N-terminus of an RNA-binding protein (ESWR1 = ES breakpoint region 1) and the carboxyl terminal DNA-binding domain of an erythroblast transformation specific (ETS) family transcription factor. In most cases the translocation t(11;22) (q24;q12) results in jointure of *ESWR1* from chromosome 22 with *FLI1* from chromosome 11 (**Figure 4**). The mechanism causing this chromosomal translocation is currently unknown. *EWSR1::FLI1* acts as a transcription factor to enable malignant transformation. Consequently, this influences the transcription of relevant downstream targets. In addition, it influences transcript degradation, alternative splicing and regulatory mechanisms for RNA abundance (6). The molecular mechanism by which the EWSR1::FLI1 fusion acts has not yet been fully characterized due to its complex nature. However, the fusion protein can act as both a transcriptional activator and a repressor. EWSR1::FLI1 can directly repress transcription by binding to wild-type ETS family binding sites in promoters and enhancers where it displaces normal transcription factors. Gene expression can also be negatively influenced indirectly by the activation of transcriptional repressors and by epigenetic mechanisms. Activation of gene expression by EWSR1::FLI1 occurs via the formation of new active gene enhancers at genomic GGAA microsatellite motifs. The recruitment of histone acetyl/methyltransferases increases chromatin accessibility and leads to the transcription of genes in previously epigenetically silenced regions of the genome (21). Although this fusion gene is tumor specific and seems to be a suitable drug target, there is no therapeutic agent approved by the Food and Drug Administration (FDA) or the European Medicines Agency (EMA) that addresses this specific defect. The complex structural features combined with the process of transcription are the main challenges for the development of small molecule inhibitors of *EWS/FLI1* (22).

In addition, a mutation of the *TERT* gene was detected with NGS. Typically, within an aging organism the telomers are shortened during the cell cycle. *TERT* allows lengthening of the telomers and thereby continuous cell division. During carcinogenesis mutations of *TERT* can be acquired that result in an infinite capacity for replication. The *TERT* activity has been found to be crucial for cell transformation. The telomerase activity is influenced by regulation at different stages including gene transcription and mRNA splicing (23). The mutation influencing *TERT* activity detected in this patient is located in the *TERT* promoter region (c.-124C>T). This alteration results in an enhanced *TERT* promotor activity by the generation of a binding site for erythroblast transformation specific (ETS) transcription factors. The *ESWR1:FLI1* fusion gene can potentially influence *TERT* activity via the binding site for ETS (24).

## 3 Discussion

This case report illustrates important aspects of the diagnosis and treatment of pulmonary Ewing sarcoma. The patient had no relevant medical history or family history of cancer. The initial symptoms leading to medical referral and diagnostic investigation were indistinct. The first histopathological examination of a transthoracic biopsy led to a diagnosis of lung cancer. Due to the severity of the symptoms and the size of the pulmonary mass chemotherapy was started before the results were obtained from the reference pathology laboratory. However, further examination of the tumor tissue in a certified reference pathology laboratory revealed a Ewing sarcoma. After diagnosis the patient was referred to our university hospital specialized and certified in sarcoma care.

The change of the initial diagnosis highlights the importance of ensuring the appropriate information is available in a timely manner for correct diagnosis and subsequent treatment. Indeed, molecular pathological confirmation, that is part of the diagnostic algorithm in specialized cancer centers, is indispensable for the confirmation of Ewing sarcoma. This further underlines the importance of such centers as molecular pathology is essential to differentiate tumors which can clinically present similarly and may need special knowledge to be correctly identified. In this case NGS allowed the identification of the *ESWR1::FLI1* gene fusion and thereby resulted in the diagnosis of a pulmonary Ewing sarcoma.

Up to now, there are no worldwide international guidelines available for the treatment of Ewing sarcomas. Nevertheless, in Europe a guideline does exist for the treatment of Ewing Sarcoma (25). Over the past decades, two main chemotherapeutic protocols have been established. In the US chemotherapy consists of an induction therapy with VDC and IE followed by consolidation therapy with alternating cycles of IE and VC (18). In Europe induction chemotherapy with VIDE is followed by consolidation chemotherapy according to risk (VAI or VAC) (17). A recent clinical trial (EE2012) found treatment with VDC and IE to be superior to VIDE and VAC/VAI. The results indicated a higher effectivity (for both event-free survival and overall survival) and reduced toxicity for VDC plus IE chemotherapy compared to VIDE and VAC/VAI (15). In accordance with these findings our patient underwent induction chemotherapy with VDC and IE. The patient displayed only mild side effects even though the patient was over 50 years of age. The resulting thrombocytopenia and leukopenia being the main side effects led to a reduction of the dose of chemotherapy used.

In some cases, chemotherapy can be followed by high-dose chemotherapy and autologous blood stem-cell rescue (17). Patients with low histological response at a localized stage may respond to this treatment. In a metastasized setting high-dose chemotherapy followed by autologous blood stem-cell rescue is not used as first-line treatment. However, in a relapsed situation this therapeutic approach is more widely used.

The EE2012 trial included patients aged 2-49 years with the median age being 15 years (15). Patients mostly present in their second to third decade of life (3, 4). The patient described in this case report was 55 years of age when admitted to our hospital for treatment of Ewing sarcoma. Thus, he was older than the average person diagnosed with Ewing sarcoma and older than any patient treated in the AEWS0031 and EE2012 trials. Nevertheless, it is important to note that the VDC plus IE chemotherapy was well tolerated and elicited a good clinical response in this older patient.

After completion of the thoracic radiotherapy the patient will be subject to regular after-care with CT scans in 3-month intervals. Ideally, these follow-up appointments should be performed at a certified sarcoma center which is crucial for early detection of tumor relapse.

## 4 Conclusion

In summary, this case report highlights the importance of a rigorous and timely histopathological examination of biopsy samples by a specialized cancer center to enable a correct diagnosis of the cancer type. Additionally, targeted NGS and RNA fusion panel sequencing plays a crucial part in this analysis and allows the necessary differentiation between cancer types, in this case between NSCLC and pulmonary Ewing sarcoma.

Currently, there is no worldwide international treatment guideline available for the treatment of Ewing sarcoma. Therefore, it is imperative to incorporate recently published international clinical data when choosing the best therapeutic approach for Ewing sarcoma. Patients should be referred to specialist centers to allow the best possible treatment of the cancer type. In the case of Ewing sarcoma and in accordance with the most recent research, patients should be treated with VDC plus IE because it has been demonstrated to be the most effective therapy. Moreover, the therapeutic regimen is well tolerated by both younger and older patients.

## Data Availability

The original contributions presented in the study are included in the article/**Supplementary Material**. Further inquiries can be directed to the corresponding author.

## Supplementary material

Supplementary Table 1 Provides a full gene list and list of biomarkers routinely analyzed and reported within the used NGS panel.
