# Supplementary material for: Case report: Pulmonary Ewing sarcoma disguised as non-small cell lung cancer
Source: Front Oncol. 2024 Nov 7;14:1449119. doi: 10.3389/fonc.2024.1449119 (PMC11578818; doi:10.3389/fonc.2024.1449119)
Supplement: Supplementary Table 1 — Provides a full gene list and list of biomarkers routinely analyzed and reported within the used NGS panel. [file DataSheet1.docx]

Supplementary Material

# Panel Overview

The following custom panel (Zentrum für Humangenetik, Tübingen "TUM version 7") was used to perform molecular genetic diagnostics based on tumor-DNA from FFPE material. The basis of the analysis is a tumor versus normal (e.g. blood) comparison. SNV/Small Indel, copy number changes, gene fusions and other biomarkers as listed below are detected as part of the analysis.

**Gene list** (detection of SNV/Small Indel and CNV)

AAK1, ABCB1, ABCG2, ABL1, ABL2, ABRAXAS1, ACD, ACVR1, ADGRA2, ADRB1, ADRB2, AIP, AIRE, AJUBA, AKT1, AKT2, AKT3, ALK, ALOX12B, AMER1, ANKRD26, APC, APLNR, APOBEC3A, APOBEC3B, AR, ARAF, ARHGAP35, ARID1A, ARID1B, ARID2, ARID5B, ASXL1, ASXL2, ATM, ATR, ATRX, AURKA, AURKB, AURKC, AXIN1, AXIN2, AXL, B2M, BAP1, BARD1, BAX, BCHE, BCL10, BCL11A, BCL11B, BCL2, BCL3, BCL6, BCL9, BCL9L, BCOR, BCORL1, BCR, BIRC2, BIRC3, BIRC5, BLM, BMI1, BMPR1A, BRAF, BRCA1, BRCA2, BRD3, BRD4, BRD7, BRIP1, BTK, BUB1B, CALR, CAMK2G, CARD11, CASP8, CBFB, CBL, CBLB, CBLC, CCDC6, CCND1, CCND2, CCND3, CCNE1, CD274, CD79A, CD79B, CD82, CDC73, CDH1, CDH11, CDH2, CDH5, CDK1, CDK12, CDK4, CDK5, CDK6, CDK8, CDKN1A, CDKN1B, CDKN1C, CDKN2A, CDKN2B, CDKN2C, CEBPA, CENPA, CEP57, CFTR, CHD1, CHD2, CHD4, CHEK1, CHEK2, CIC, CIITA, CKS1B, CNKSR1, COL1A1, COMT, COQ2, CREB1, CREBBP, CRKL, CRLF2, CRTC1, CSF1R, CSF3R, CSMD1, CSNK1A1, CTCF, CTLA4, CTNNA1, CTNNB1, CTR9, CTRC, CUX1, CXCR4, CYLD, CYP1A2, CYP2A7, CYP2B6, CYP2C19, CYP2C8, CYP2C9, CYP2D6, CYP3A4, CYP3A5, CYP4F2, DAXX, DCC, DDB2, DDR1, DDR2, DDX11, DDX3X, DDX41, DEK, DHFR, DICER1, DIS3L2, DNMT1, DNMT3A, DOT1L, DPYD, E2F3, EBP, EED, EFL1, EGFR, EGLN1, EGLN2, EIF1AX, ELAC2, ELF3, EME1, EML4, EMSY, EP300, EPAS1, EPCAM, EPHA2, EPHA3, EPHB4, EPHB6, ERBB2, ERBB3, ERBB4, ERCC1, ERCC2, ERCC3, ERCC4, ERCC5, ERG, ERRFI1, ESR1, ESR2, ETNK1, ETV1, ETV4, ETV5, ETV6, EWSR1, EXO1, EXT1, EXT2, EZH1, EZH2, FAN1, FANCA, FANCB, FANCC, FANCD2, FANCE, FANCF, FANCG, FANCI, FANCL, FANCM, FAS, FAT1, FBXO11, FBXW7, FEN1, FES, FGF10, FGF14, FGF19, FGF2, FGF23, FGF3, FGF4, FGF5, FGF6, FGF9, FGFBP1, FGFR1, FGFR2, FGFR3, FGFR4, FH, FLCN, FLI1, FLT1, FLT3, FLT4, FOXA1, FOXE1, FOXL2, FOXO1, FOXP1, FOXQ1, FRK, FRS2, FUBP1, FUS, FYN, G6PD, GALNT12, GATA1, GATA2, GATA3, GATA4, GATA6, GGT1, GLI1, GLI2, GLI3, GNA11, GNA13, GNAQ, GNAS, GNB3, GPC3, GPER1, GREM1, GRIN2A, GRM3, GSK3A, GSK3B, GSTP1, H3-3A, H3-3B, H3C2, HABP2, HCK, HDAC1, HDAC2, HDAC6, HGF, HIF1A, HLA-A, HLA-B, HLA-C, HLA-DPA1, HLA-DPB1, HLA-DQA1, HLA-DQB1, HLA-DRA, HLA-DRB1, HMGA2, HMGCR, HMGN1, HNF1A, HNF1B, HOXB13, HRAS, HSD3B1, HSP90AA1, HSP90AB1, HTR2A, ID2, ID3, IDH1, IDH2, IDO1, IFNGR1, IFNGR2, IGF1R, IGF2, IGF2R, IKBKB, IKBKE, IKZF1, IKZF3, IL1B, IL1RN, ING4, INPP4A, INPP4B, INPPL1, INSR, IRF1, IRF2, IRS1, IRS2, ITPA, JAK1, JAK2, JAK3, JUN, KAT6A, KDM5A, KDM5C, KDM6A, KDR, KEAP1, KIAA1549, KIF1B, KIT, KLF2, KLF4, KLHL6, KLLN, KMT2A, KMT2B, KMT2C, KMT2D, KRAS, KSR1, LATS1, LATS2, LCK, LIG4, LIMK2, LRP1B, LRRK2, LTK, LYN, LZTR1, MAD2L2, MAF, MAGI1, MAGI2, MAML1, MAP2K1, MAP2K2, MAP2K3, MAP2K4, MAP2K5, MAP2K6, MAP2K7, MAP3K1, MAP3K13, MAP3K14, MAP3K3, MAP3K4, MAP3K6, MAP3K8, MAPK1, MAPK11, MAPK12, MAPK14, MAPK3, MAX, MBD1, MBD4, MC1R, MCL1, MDC1, MDH2, MDM2, MDM4, MECOM, MED12, MEF2B, MEN1, MERTK, MET, MGA, MGMT, MITF, MLH1, MLH3, MLLT10, MLLT3, MN1, MPL, MRE11, MS4A1, MSH2, MSH3, MSH4, MSH5, MSH6, MSR1, MST1R, MTAP, MTHFR, MTOR, MT-RNR1, MTRR, MUC1, MUTYH, MXI1, MYB, MYC, MYCL, MYCN, MYD88, MYH11, MYH9, NAT2, NBN, NCOA1, NCOA3, NCOR1, NF1, NF2, NFE2L2, NFKB1, NFKB2, NFKBIA, NFKBIE, NIN, NKX2-1, NLRC5, NOTCH1, NOTCH2, NOTCH3, NOTCH4, NPM1, NQO1, NR1I3, NRAS, NRG1, NSD1, NSD2, NSD3, NT5C2, NTHL1, NTRK1, NTRK2, NTRK3, NUMA1, NUP98, NUTM1, OBSCN, OPRM1, PAK1, PAK3, PAK4, PALB2, PALLD, PARP1, PARP2, PARP4, PAX3, PAX5, PAX7, PBK, PBRM1, PBX1, PDCD1, PDCD1LG2, PDGFA, PDGFB, PDGFC, PDGFD, PDGFRA, PDGFRB, PDK1, PDPK1, PGR, PHF6, PHOX2B, PIAS4, PIGA, PIK3C2A, PIK3C2B, PIK3C2G, PIK3CA, PIK3CB, PIK3CD, PIK3CG, PIK3R1, PIK3R2, PIK3R3, PIM1, PLCG1, PLCG2, PLK1, PML, PMS1, PMS2, POLD1, POLE, POLH, POLQ, POT1, PPM1D, PPP2R1A, PPP2R2A, PREX2, PRKAR1A, PRKCA, PRKCI, PRKDC, PRKN, PRMT5, PRSS1, PSMB1, PSMB10, PSMB2, PSMB5, PSMB8, PSMB9, PSMC3IP, PSME1, PSME2, PSME3, PSPH, PTCH1, PTCH2, PTEN, PTGS2, PTK2, PTK7, PTPN11, PTPN12, PTPRC, PTPRD, PTPRS, PTPRT, RABL3, RAC1, RAC2, RAD21, RAD50, RAD51, RAD51B, RAD51C, RAD51D, RAD54B, RAD54L, RAF1, RALGDS, RARA, RASA1, RASAL1, RB1, RBM10, RECQL4, REST, RET, RFC2, RFWD3, RFX5, RFXANK, RFXAP, RHBDF2, RHEB, RHOA, RICTOR, RINT1, RIPK1, RIT1, RNASEH2B, RNASEL, RNF43, ROS1, RPS20, RPS6KB1, RPS6KB2, RPTOR, RSF1, RUNX1, RYR1, SAMHD1, SAV1, SBDS, SCG5, SDHA, SDHAF2, SDHB, SDHC, SDHD, SEC23B, SERPINB9, SETBP1, SETD2, SETDB1, SF3B1, SGK1, SH2B1, SH2B3, SHH, SIK2, SIN3A, SKP2, SLC19A1, SLC26A3, SLCO1B1, SLIT2, SLX4, SMAD3, SMAD4, SMARCA4, SMARCB1, SMARCE1, SMC1A, SMC3, SMO, SOCS1, SOS1, SOX11, SOX2, SOX9, SPEN, SPINK1, SPOP, SPRED1, SRC, SRD5A2, SRGAP1, SRSF2, SSTR2, SSX1, STAG1, STAG2, STAT1, STAT3, STAT5A, STAT5B, STK11, SUFU, SUZ12, SYK, TAF1, TAF15, TAP1, TAP2, TAPBP, TBK1, TBL1XR1, TBX3, TCF3, TCF4, TCL1A, TEK, TERC, TERF2IP, TERT, TET1, TET2, TFE3, TGFB1, TGFBR2, TMEM127, TMPRSS2, TNFAIP3, TNFRSF13B, TNFRSF14, TNFRSF8, TNFSF11, TNK2, TOP1, TOP2A, TP53, TP53BP1, TP63, TPMT, TPX2, TRAF2, TRAF3, TRAF5, TRAF6, TRAF7, TRIM28, TRRAP, TSC1, TSC2, TSHR, TTK, TYMS, U2AF1, UBE2T, UBR5, UGT1A1, UGT2B15, UGT2B7, UIMC1, UNG, USP9X, VEGFA, VEGFB, VHL, VKORC1, WRN, WT1, XIAP, XPA, XPC, XPO1, XRCC1, XRCC2, XRCC3, XRCC5, XRCC6, YAP1, YES1, ZFHX3, ZNF217, ZNF703, ZNRF3, ZRSR2 (749 Genes)

*DPYD*, *G6PD*, *RYR1*, *TPMT*, *UGT1A1* (Pharmacogenetics)

**Biomarker list** **/ secondary analysis** (analysis derived from NGS panel data)

Homologous recombination deficiency (HRD), Microsatellite instability (MSI/MSS), Tumor mutational burden (TMB), viral infection (HPV/EBV)

**Structural variants** (gene fusions, selected breakpoints) potentially affecting the following genes are being assessed:

ALK, BCL2, BCR, BRAF, BRD4, EGFR, ERG, ETV4, ETV6, EWSR1, FGFR1, FGFR2, FGFR3, FUS, MET, MYB, MYC, NOTCH2, NTRK1, NTRK2, NTRK3, PAX3, PDGFB, RAF1, RARA, RET, ROS1, SSX1, SUZ12, TAF15, TCF3, TFE3, TMPRSS2

# 
